# Appraisal of systematic reviews on interventions for postpartum depression: systematic review

**DOI:** 10.1186/s12884-020-03496-5

**Published:** 2021-01-06

**Authors:** Ryan Chow, Eileen Huang, Allen Li, Sophie Li, Sarah Y. Fu, Jin S. Son, Warren G. Foster

**Affiliations:** 1 Faculty of Medicine, University of Ottawa, 451 Smyth Road, Ottawa, ON K1N 6N5 Canada; 2 Department of Obstetrics and Gynecology, HSC-3N52D, McMaster University, 1280 Main St W, Hamilton, ON L8S 4K1 Canada; 3 Faculty of Health Sciences, McMaster University, 1280 Main St W, Hamilton, ON L8S 4K1 Canada

**Keywords:** AMSTAR, Cochrane reviews, Methodological rigor, PRISMA, Mental health, women’s health

## Abstract

**Background:**

Postpartum depression (PPD) is a highly prevalent mental health problem that affects parental health with implications for child health in infancy, childhood, adolescence and beyond. The primary aim of this study was to critically appraise available systematic reviews describing interventions for PPD. The secondary aim was to evaluate the methodological quality of the included systematic reviews and their conclusions.

**Methods:**

An electronic database search of MEDLINE, Embase, and the Cochrane Library from 2000 to 2020 was conducted to identify systematic reviews that examined an intervention for PPD. *A Measurement Tool to Assess Systematic Reviews* was utilized to independently score each included systematic review which was then critically appraised to better define the most effective therapeutic options for PPD.

**Results:**

Of the 842 studies identified, 83 met the a priori criteria for inclusion. Based on the systematic reviews with the highest methodological quality, we found that use of antidepressants and telemedicine were the most effective treatments for PPD. Symptoms of PPD were also improved by traditional herbal medicine and aromatherapy. Current evidence for physical exercise and cognitive behavioural therapy in treating PPD remains equivocal. A significant, but weak relationship between AMSTAR score and journal impact factor was observed (*p* = 0.03, r = 0.24; 95% CI, 0.02 to 0.43) whilst no relationship was found between the number of total citations (*p* = 0.27, r = 0.12; 95% CI, − 0.09 to 0.34), or source of funding (*p* = 0.19).

**Conclusion:**

Overall the systematic reviews on interventions for PPD are of low-moderate quality and are not improving over time. Antidepressants and telemedicine were the most effective therapeutic interventions for PPD treatment.

**Supplementary Information:**

The online version contains supplementary material available at 10.1186/s12884-020-03496-5.

## Background

Childbirth (parturition) can cause significant change in a woman’s priorities, roles, and responsibilities. Though there are many concerns for the mother after parturition, emergence of postpartum depression (PPD) and clinical management strategies remain an important unresolved issue [1]. PPD is defined by the *Diagnostic and Statistical Manual of Mental Disorders IV* and is an increasingly prevalent mental health problem that typically begins four to six weeks after parturition [2]. Common symptoms include sleep and appetite disturbance, diminished concentration, irritability, anxiety, feelings of guilt and worthlessness, loss of interest or pleasure in daily activities, depressed mood and thoughts of suicide [3].

The prevalence of PPD differs significantly depending on the country and ranges from 1.9 to 82.1% with the highest reported prevalence appearing in the United States and the lowest in Germany [4]. The consequences of PPD on the child are not restricted to infancy, and can extend into toddlerhood, school age, and even adolescence. PPD can lead to inadequate prenatal care, childhood noncompliance, anger and dysregulated attention, and lower cognitive performance [5]. As the window to treat PPD is time-sensitive, it is critical to define the efficacy and safety of different therapeutic options. PPD is a complex disorder whose pathophysiology remains poorly defined with sub-optimal therapeutic options and an expanding literature. Numerous systematic reviews describing therapeutic interventions for the management of PPD have emerged in the literature in recent years; however, the most effective therapeutic options remain poorly defined.

Evidence-based medicine is defined as using highest-quality evidence to inform clinical decision-making [6]. In the hierarchy of evidence, systematic reviews and meta-analyses sit as the very top [7]. If done correctly, systematic reviews and meta-analyses are able to consolidate and summarize primary evidence for clinicians and policymakers. However, when systematic reviews are poorly conducted, their risk to bias increases and can generate invalid and unreliable results. Guidelines such as the *Preferred Reporting Items for Systematic Reviews and Meta-analyses* (PRISMA) and *Meta-analysis Of Observational Studies in Epidemiology* have been developed to ensure consistency in the methodological synthesis of systematic reviews [8, 9]. In addition to those, the Assessing the Methodological Quality of Systematic Reviews (AMSTAR) tool was developed and is a validated tool [10, 11] to assess the methodological quality of systematic reviews.

## Methods

The aim of this study was to evaluate the quality of systematic reviews on the efficacy and safety of different PPD interventions using the AMSTAR tool and to evaluate different therapeutic strategies stratified by methodological quality. The secondary aim was to investigate whether different publication characteristics (e.g. number of citations, impact factor of the journal, year of publication, funding source) were associated with the methodological rigour of the systematic review. This systematic review was conducted according to PRISMA guidelines [8].

### Search strategy

A comprehensive electronic database search, with a validated search strategy from a medical librarian, of Embase, MEDLINE and the Cochrane Library of Systematic Reviews from inception until March 5th, 2020 was conducted. Search terms include depression, postpartum or post-partum, postnatal or post-natal, and systematic review (Appendix S1). The complete search strategy is available in the online supplement (Table S1).

### Study selection

Search results were uploaded into the Covidence software platform (Veritas Health Innovation Ltd). Duplicate articles were removed, and a two-staged independent screening process was used to identify studies for inclusion. Pilots were run for the initial stage of screening until review authors (E.H., S.F., S.L. and J.S.) reached a Cohen’s kappa inter-rater reliability value of 0.8 [12]. Subsequently, reviewers independently screened titles and abstracts. Eligible articles proceeded to full-text screening. Discrepancies during either stage of screening were resolved by discussion among the authorship team until a consensus was reached. The inclusion criteria involved: (1) the systematic review must investigate the safety and/or effectiveness of any intervention treating post-partum depression; (2) self-identified as a systematic review in the title or abstract; (3) the systematic review must review primary literature. The exclusion criteria involved: (1) outdated reviews where an updated version was accessible; (2) systematic reviews of other systematic reviews; (3) meta-analyses that did not include a systematic review; (4) non-intervention systematic reviews (e.g. preventative or screening tools); (5) reviews aiming to investigate the state of literature, where patient outcomes were not the primary interest; (6) non-English literature, and (7) conference abstracts.

### Data extraction

Data was independently extracted by authors (E.H., S.F., S.L. and J.S.). Domains extracted included publication details such as: journal and impact factor (from Clarivate Analytics), year of publication, funding source (e.g. philanthropic, government, industry, etc.), total citations (from Google Scholar), conflict of interest statement (dichotomous), the corresponding author’s country, and the intervention studied (e.g. peer support groups, antidepressants, cognitive behavioural therapy, etc.). Discrepancies were resolved by discussion and consensus among the authorship team. The list of excluded studies is available in the online supplement (Table S2).

### Risk of Bias assessment

Authors (E.H., S.F., S.L. and J.S.) independently evaluated the methodological quality of the studies using the AMSTAR quality assessment tool. Scores were tabulated using Microsoft Excel (Redmond, Wash.). Review authors selected either “yes,” “not applicable,” “no,” or “can’t answer” for each of AMSTAR criteria. Discrepancies were resolved by consensus with the authorship team. A point was awarded for each of the AMSTAR criteria that received a “yes.” No points were given for “not applicable,” “no,” or “can’t answer”. Therefore, the highest total score possible was 11.

### Strategy for data synthesis

Tables generated using Microsoft Excel (Redmond, Wash.) were used to summarize data. GraphPad Prism (version 7.0, GraphPad Software, Inc., USA) was used to statistically analyze data. Pairwise correlations (AMSTAR Score vs. Total Citations, AMSTAR Score vs. Impact Factor, AMSTAR Score vs. Publication Year) were evaluated using the Pearson correlation coefficient (r). A two-tailed T-Test was used to evaluate potential differences in AMSTAR Score in terms of source of funding (Cochrane article vs. non-Cochrane article, government vs. institution etc.). A *P*-value less than 0.05 was considered statistically significant.

Included studies were stratified into low, moderate, and high methodological quality, as identified by an AMSTAR score of 1–5, 6–8, and 9–11, respectively (Table S3). Findings from included studies were then narratively synthesized within each stratum. Greater emphasis was placed on extensively researched interventions or reviews with greater methodological rigor.

## Results

### Study selection

**Fig. 1:**
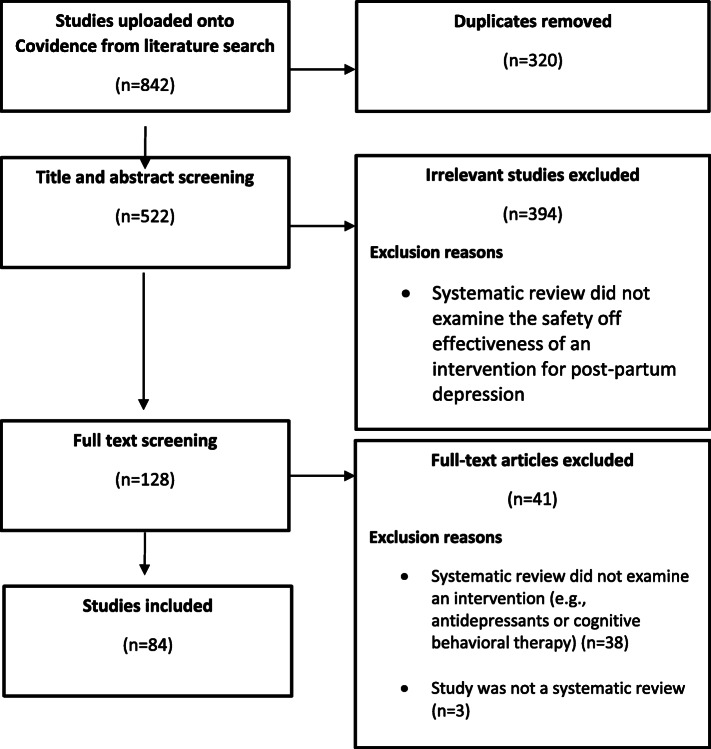
Flow Diagram illustrating the management of article titles identified in our literature search, rationale for study exclusion and ultimate inclusion for critical appraisal

The electronic searches identified 842 publications, of which 320 (38%) were duplicates (Fig. 1). 522 articles proceeded to title/abstract screening with 394 (47%) being deemed ineligible as they did not evaluate an intervention for PPD. 128 (17%) full-text articles were retrieved and subjected to another round of screening from which 41 (5%) studies were excluded as they did not examine interventions for PPD. Three (0.3%) more studies were excluded as they were not systematic reviews. Finally, 84 studies (10%) met the a priori inclusion/exclusion criteria and were included [13–95] for critical appraisal.

### Characteristics of included studies

The characteristics of included studies are described in Table S1. The greatest number of studies (*n* = 15; 18%) were published in the Cochrane Library with the second most (*n* = 6; 7%) published in The Archives of Women’s Health. Institutional funding involving hospitals and universities were involved with the largest proportion of studies (*n* = 28; 33%). Government sources of funding were involved in a minority of publications (*n* = 17; 20%), no funding was reported for (*n* = 20; 24%) articles, and many articles failed to report a funding source (*n* = 25; 30%) (Table 1).

**Table 1:** Characteristics of Funding and Origin of Included Systematic Reviews

*Publication frequency based on country*	
Country of Corresponding Author	Number of publications
United Kingdom	14
Canada	12
Australia	11
United States	10
England	9
China	7
Japan	3
Italy	3
France	2
Thailand	1
Taiwan	1
Singapore	1
Scotland	1
Romania	1
Portugal	1
Poland	1
Pakistan	1
Korea	1
Ireland	1
Iran	1
Germany	1
Brazil	1
*Funding of studies*	
**Source of Funding**	**Number of studies**
Government	10
Institution	5
Government and Philanthropic or Institution	7
None	20
None reported	25

Of the different therapeutic interventions described, peer support and group therapy were the intervention most frequently examined (n = 20; 24%), whereas cognitive behavioural therapy (CBT) and physical activity were less frequently examined (*n* = 17, 20%; *n* = 10 = 12%, respectively) of the studies reviewed. Some interesting interventions such as skin-to-skin infant contact, hypnosis, and specific traditional rituals were only reported in a single systematic review.

### Methodological quality of included studies

**Fig. 2:**
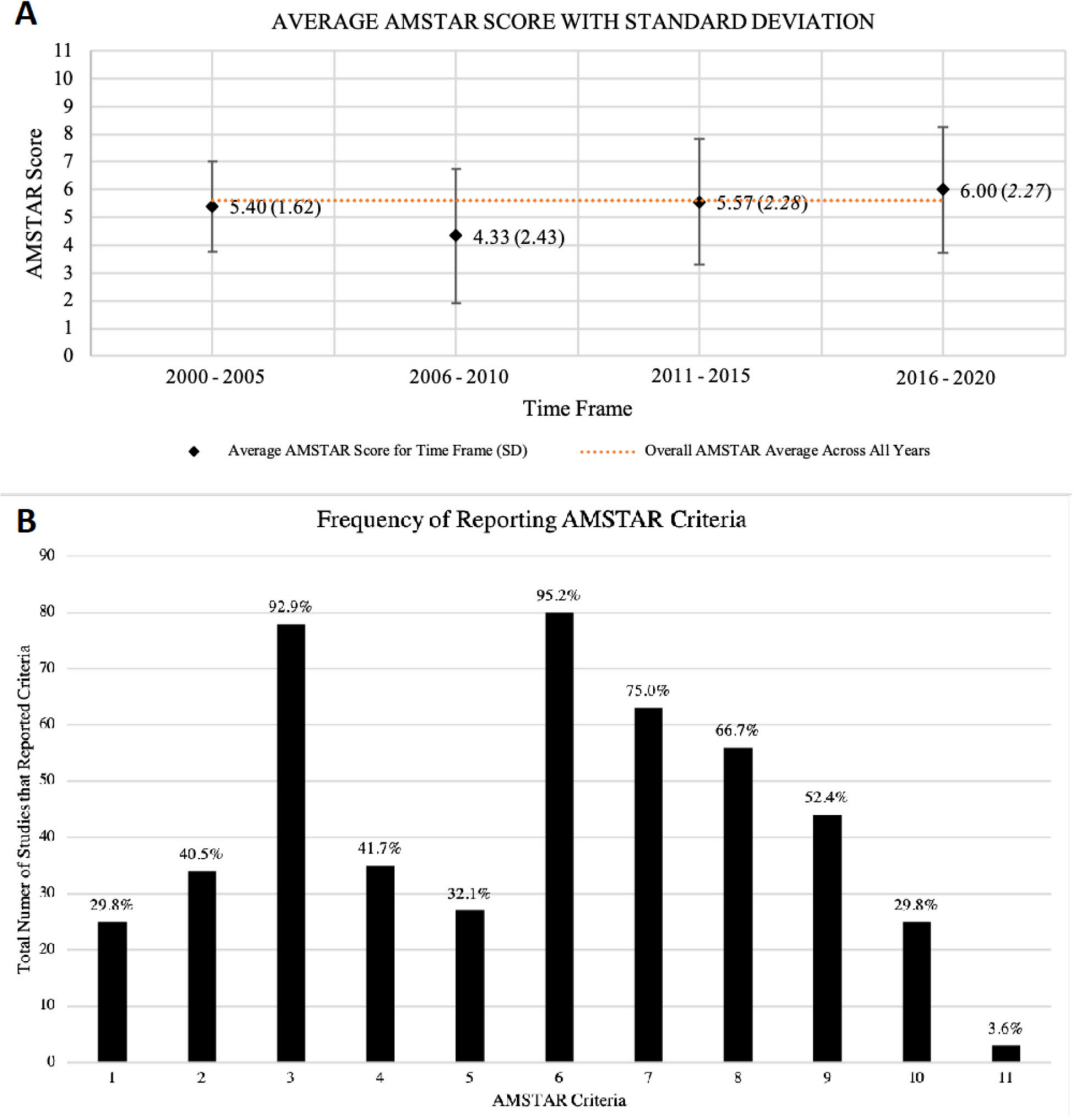
The Characteristics of the AMSTAR Assessment of Included Studies. A: The AMSTAR scores from 2000 to 2020, grouped into five-year intervals. Data represented as mean (SD). The mean AMSTAR score throughout the past twenty years was 5.6 (1.6). B: The number (%) of studies adhering to each AMSTAR criteria. Criteria: 1. Was an ‘a priori’ design provided? 2. Was there duplicate study selection and data extraction? 3. Was a comprehensive literature search performed? 4. Was the status of publication (i.e. grey literature) used as an inclusion criterion? 5. Was a list of studies (included and excluded) provided? 6. Were the characteristics of the included studies provided? 7. Was the scientific quality of the included studies assessed and documented? 8. Was the scientific quality of the included studies used appropriately in formulating conclusions? 9. Were the methods used to combine the findings of studies appropriate? 10. Was the likelihood of publication bias assessed? 11. Was the conflict of interest included?

The overall AMSTAR score for included studies published from 2000 to 2020 had a mean (SD) of 5.6 ± 1.6 (Fig. 2a). Compliance to each AMSTAR criteria was inconsistent across the studies (Fig. 2b). The overall methodological quality of the systematic reviews assessed was highly variable, with AMSTAR scores ranging from 1/11 (*n* = 5; 6%) to 10/11 (*n* = 2; 2.4%). The top three AMSTAR criteria that were most satisfied involved inclusion of the characteristics of included studies (criterion 6: *n* = 80; 95.2% of studies), the performance of a comprehensive literature search (criterion 3: *n* = 78; 92.9% of studies), and the inclusion of a quality assessment (criterion 7: *n* = 63; 75% of studies). The three AMSTAR criteria that were the least frequently reported were the reporting of funding sources of included studies (criterion 11: *n* = 3; 3.6% of studies), and a tie between an a priori design and the assessment for publication bias (criterion 1 and 10: *n* = 25; 29.8% of studies), and the reporting of the included and excluded studies (criterion 5: *n* = 27; 32.1% of studies).

### Synthesis of results

**Fig. 3:**
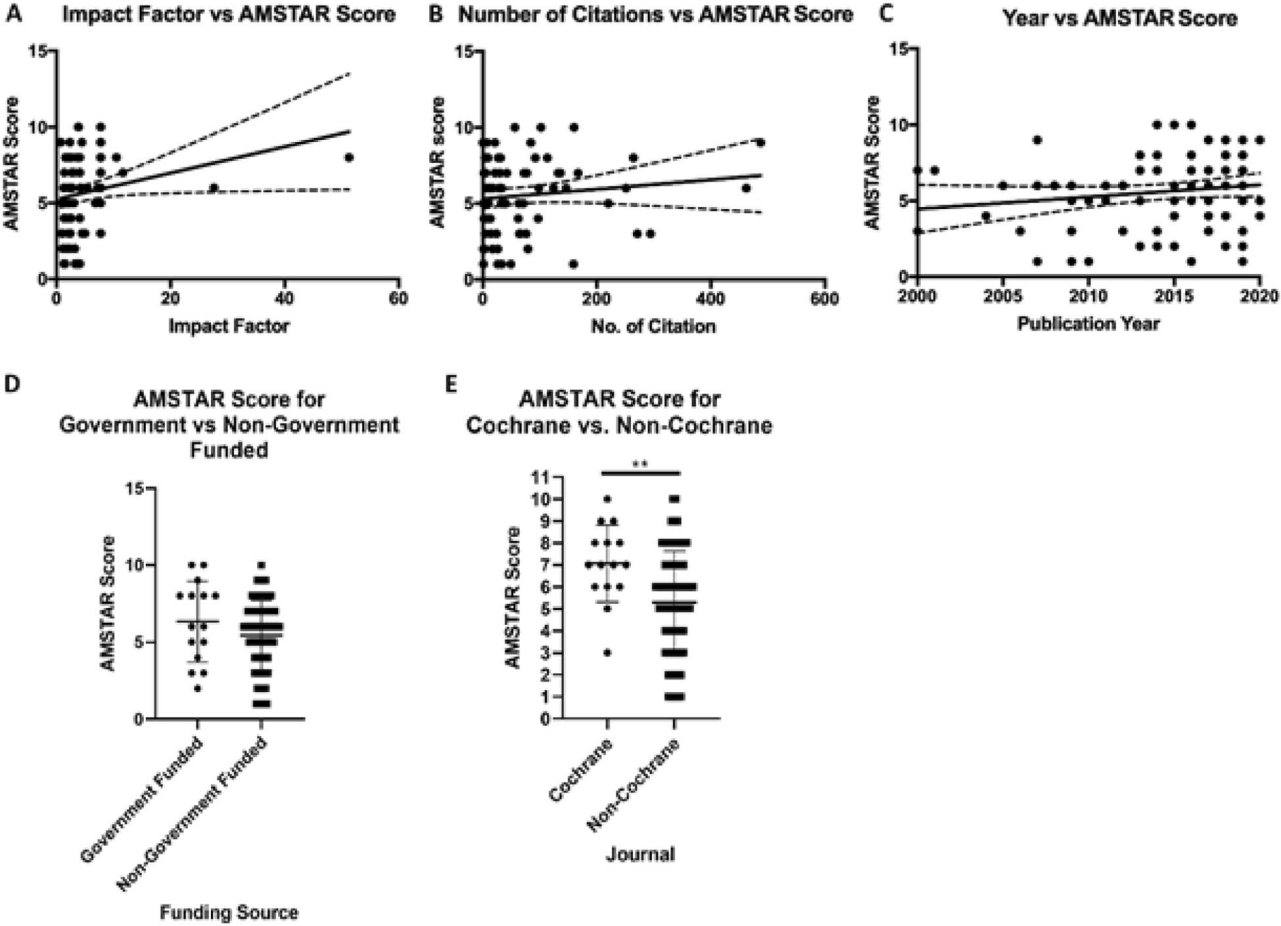
Association Between Publication Factors and Methodological Quality. A: AMSTAR score vs. journal impact factor (*p* = 0.03, r = 0.24; 95% CI, 0.02 to 0.43). B: AMSTAR score vs. number of citations (*p* = 0.27, r = 0.12; 95% CI, − 0.09 to 0.34). C: AMSTAR score vs. publication year (*p* = 0.14, r = 0.16; 95% CI, − 0.05 to 0.37). D: Differences in AMSTAR score in papers funded by government vs. non-government sources. (*p* = 0.18) E: Differences in AMSTAR score for papers published by the Cochrane Collaboration vs. published in non-Cochrane journals (**p = 0.007)

Almost half of the included systematic reviews were of low quality (*n* = 37) as identified by an AMSTAR score of 1–5. A significant, but weak relationship between AMSTAR score and journal impact factor was observed (Fig. 3a; *p* = 0.03, r = 0.24; 95% CI, 0.02 to 0.43). No significant relationships between mean AMSTAR score and number of citations (Fig. 3b; *p* = 0.27, r = 0.12; 95% CI, − 0.09 to 0.34) or publication year (Fig. 3c; *p* = 0.14, r = 0.16; 95% CI, − 0.05 to 0.37) were found. No significant differences (*p* = 0.19) were found between the AMSTAR scores of systematic reviews funded by government funding agencies, philanthropists, or institutions (Fig. 3d). On average, systematic reviews published by the Cochrane Collaboration scored higher than other published systematic reviews we evaluated (*p* = 0.007) (Fig. 3e).

For the most highly ranked systematic reviews, the most common interventions studied evaluated involved traditional interventions such as aromatherapy, acupuncture, and rituals [27, 85, 88, 89], as well as more conventional therapies such as CBT [30, 39, 95], physical activity [34, 65, 81], and pharmacological treatments [29, 42, 44, 55, 57, 79]. Positive benefits of aromatherapy on PPD were reported in two [85, 89] systematic reviews, but meta-analysis was not possible due to the heterogeneity of study designs therein. A systematic review on acupuncture reported a pooled mean difference of − 1.27 (95% CI, − 2.55 to 0.01; *p* = 0.05, I^2^ = 83%) on the Hamilton Depression Scale between 12 randomized controlled trials (RCTs) with 887 participants [88]. However, they reported that the trials included therein had a high risk of bias and that future trials with higher methodological rigour would be needed to confirm the beneficial effects of acupuncture. Finally, there was no clear evidence on of a beneficial effect of traditional rituals on PPD. [27]

The efficacy of cognitive behaviour therapy (CBT) as a PPD intervention was examined by multiple reviews. CBT reduced *Edinburgh Postnatal Depression Scale* (EPDS) depression scores in a meta-analysis of six studies (− 4.48, 95% CI, 1.01 to 7.95) [30]. Another meta-analysis of seven RCTs showed a significant size-effect of CBT on reducing PPD (d = − 0.54, 95% CI, − 0.716; − 0.423) [95]. However, a third systematic review found inconsistent and inconclusive results regarding its effectiveness [39]. Thus, the benefits of CBT as a therapeutic option for the management of PPD remain to be clarified. It is important to note that primary studies and trials with significant limitations were used to reach these conclusions.

In the present review, most of the included systematic reviews were ranked as moderate quality (*n* = 39), characterized by an AMSTAR score of 6–8. About a fourth of the studies in this stratum were published in the Cochrane Database of Systematic Reviews (*n* = 10) and most of the reviews were either funded by institutions (*n* = 15) or did not receive financial support (*n* = 12). The most extensively researched interventions in this stratum were also traditional interventions. Results of a meta-analysis of seven RCTs demonstrated that Chai Hu Shu Gan San had a greater effect on postpartum depression (mean difference = − 4.10, 95% CI, − 7.48 to − 0.72, I^2^ = 86%) compared to fluoxetine [76]. Another systematic review also stated that other forms of Chinese herbal medicine could reduce depression scores, alone or in combination with routine treatments [53, 77]. Taken together these data suggest that traditional Chinese herbal medicine could have beneficial effects in the treatment of PPD and provide a useful alternative therapeutic option for women preferring natural therapies over conventional options.

Pharmacological interventions, including antidepressants and hormonal treatments, were also extensively researched [1, 14, 43, 46, 87]. Estrogen therapy, progestin-only pills, and levonorgestrel intrauterine devices were reported to be effective, but a limited number of trials were referenced [87]. On the contrary, another systematic review reported [24] that in a double-blind randomised placebo-controlled trial, norethisterone enanthate increased the risk of developing PPD (mean EPDS score 10.6 vs 7.5; *P* = 0.0022). Three systematic reviews reported that fluoxetine [14, 43, 46] is an effective therapeutic option for PPD. Fluoxetine decreased EPDS depression scores from (9.9 (8.3 to 11.8)] to [7.3 (5.5 to 9.6)) compared to placebo, in a trial with 87 women [14]. It is reported that most included trials from these systematic reviews were indicated to have a high risk of bias and that results should be interpreted with caution [46].

The effectiveness of telephone support as a PPD intervention was investigated in three systematic reviews [22, 23, 37]. Findings of one study found that telephone support delivered by health professionals was associated with lower depression scores in the postnatal period [37]. Telephone peer support was examined in a systematic review that included seven trials with 2492 participants. They found that telephone peer support significantly reduced depressive symptomatology, as rated by the EPDS, at eight weeks postpartum (OR 6.23, 95% CI, 1.40 to 27.84; *P* = 0.01) [22]. However, the methods of administering peer telephone support from the primary studies remain unclear. Additionally, evidence from another systematic review of five primary studies showed an average reduction in EPDS scores of 3.02 (95% CI, 5.34 to 0.70) [73]. Based on these systematic reviews with a fair rating of methodological rigour, telecommunication strategies show promise as an effective intervention for patients with PPD.

Physical exercise was another extensively researched intervention. A systematic review conducted a robust variance estimation random-effects meta-analysis and found a significant reduction in postpartum depression scores (Overall standard mean difference (SMD) = − 0.22 (95% CI, − 0.42 to − 0.01), *p* = 0.04; I^2^ = 86.4%) in women physically active during pregnancy relative to those who were not [83]. Another systematic review found that exercise reduced women’s PPD, as reported by the EPDS, by − 4.00 points (95% CI, − 7.64 to − 0.35) [26]. These findings were contrary to a systematic review that did not find exercise to reduce postnatal depressive symptoms [68]. It is evident that studies with greater methodological rigour must be conducted to determine the effectiveness of physical exercise as an intervention for PPD.

The highest AMSTAR score achieved was 10/11 (*n* = 2) and involved a paper published in the Journal of Epidemiology and Community Health, and another in the Cochrane Database of Systematic Reviews. One of these systematic reviews analyzed the use of conventional pharmacological antidepressants [46], whereas another examined the role of male involvement [52]. Important conclusions from these studies include that selective serotonin re-uptake inhibitors (SSRIs) such as sertraline, paroxetine and fluoxetine have been shown to have a positive impact in mother’s experiencing PPD (response: RR 1.43, 95% CI, 1.01 to 2.03); remission: RR 1.79, 95% CI, 1.08 to 2.98). Furthermore, a conventional tricyclic antidepressant, nortriptyline, was equally as effective as sertraline. It was concluded that there was no meaningful difference in adverse effects between treatment arms in the studies included in the systematic reviews, although very limited data on effects experienced by breastfed infants were available. Another study [52] reported that male involvement during antenatal care was associated with a greater utilization of healthcare services and higher quality postnatal care (OR = 1.35, 95% CI not reported; *p* = 0.01). Male involvement in the post-partum period significantly decreased the likelihood of PPD by 66% (OR 0.34, 95% CI, 0.19 to 0.62; I^2^ = 57%).

## Discussion

Overall, our results revealed a low-moderate level of methodological quality with no statistically significant changes in quality over the past 20 years. Use of antidepressants and telecommunication therapy were the most effective interventions for PPD based on the systematic reviews with the highest methodological quality. In addition, traditional Chinese herbal medicine was also found to be an effective tool for the treatment of PPD and thus may serve as a useful treatment alternative for women who prefer natural therapies over conventional methods. The use of physical exercise, hormonal therapies, and CBT for the treatment of PPD remain equivocal.

There was a weak but significant correlation observed between AMSTAR score and the impact factor of the journal, suggesting that leading journals may evaluate methodological quality a little more rigorously than others. Given the overall low-moderate quality of systematic reviews, it would be beneficial for editorial boards to integrate quality assessment tools in the peer review process. Furthermore, there was no significant correlation between AMSTAR score and total number of citations an article had. This is an observation that is consistently seen in other realms such as hematology [96].

Systematic reviews published by the Cochrane Library had an average score that was higher compared to non-Cochrane articles (*p* = 0.007). This observation supports the generally accepted position that the Cochrane Collaboration sets a high standard for methodological rigour when undertaking systematic reviews. These results align with the findings from other medical disciplines regarding the methodological quality of Cochrane reviews as well [97].

A large level of heterogeneity was observed in the quality assessment of peer-reviewed systematic reviews involving the safety and effectiveness of pharmacological and psychosocial interventions to treat PPD. AMSTAR scores ranged from 1/11 (*n* = 5) to 10/11 (*n* = 2). The number of systematic reviews in this field has slowly increased over the past two decades, with the most (*n* = 14) being published in 2019. However, our evaluation of systematic reviews (*n* = 83) did not detect improvements in methodological rigour over the last two decades. This finding diverges from other areas in research, like radiology and critical care, in which methodological rigour of systematic reviews has improved over time [98, 99].

A strength of the present study is that a comprehensive literature search according to the AMSTAR criteria was conducted and the PRISMA statement was adhered to. A large scope of evidence was available and retrieved from the Cochrane Library, Medline, and Embase. One limitation of our study is that the quality of the systematic reviews evaluated was carried out by authors aware of the authorship and publication journal of the study. However, the potential for bias was reduced by several authors independently evaluating each systematic review, with final decisions for each quality assessment criteria followed by discussion until consensus was achieved. Furthermore, the analysis between AMSTAR score and the number of citations may be affected by publication date of the systematic review. Recently published systematic reviews may not have garnered as many citations as older publications, even if AMSTAR scores may be higher. However, we utilized this metric as it provides insight on how the methodological quality of given systematic reviews have influenced the field.

## Conclusions

The methodological rigor of the systematic reviews of therapeutic options for women with PPD over the past 20 years is of low to moderate quality and has remained unchanged over time. We found that, based on the systematic reviews with the highest methodological quality, the use of antidepressants and telecommunication therapy are the most effective interventions for PPD. Traditional Chinese herbal medicine was effective in the management of PPD and thus could provide a useful therapeutic alternative for women who prefer natural options over conventional therapies. The efficacy of physical exercise, hormonal therapies, and CBT for the treatment of PPD remain equivocal.

## Supplementary Information

**Additional file 1.** Table S1: Included Studies and Their Characteristics. Table S2: List of Excluded Studies and Their Reasons. Table S3: AMSTAR Scoring of Included Studies. Appendix S1: Search Keywords and Search Strings.

## Data Availability

All data generated or analysed during this study are included in this published article and its supplementary information files.

## Abbreviations

AMSTAR: *A Measurement Tool to Assess Systematic Reviews*
CBT: Cognitive behavioural therapy
EPDS: Edinburgh Postnatal Depression Scale
PPD: Post-partum depression
