# Supplementary material for: Appraisal of systematic reviews on interventions for postpartum depression: systematic review
Source: BMC Pregnancy Childbirth. 2021 Jan 6;21:18. doi: 10.1186/s12884-020-03496-5 (PMC7789727; doi:10.1186/s12884-020-03496-5)
Supplement: Supplementary file 1 — Additional file 1. Table S1: Included Studies and Their Characteristics. Table S2: List of Excluded Studies and Their Reasons. Table S3: AMSTAR Scoring of Included Studies. Appendix S1: Search Keywords and Search Strings. [file 12884_2020_3496_MOESM1_ESM.docx]

**Supplemental Material**

Table S1: Included Studies and Their Characteristics Pg. 1-7

Table S2: List of Excluded Studies and Their Reasons Pg. 7-9

Table S3: AMSTAR Scoring of Included Studies Pg. 9-11

*Appendix S1: Search Keywords and Search Strings* Pg. 8

Table S1: Included Studies and Their Characteristics

| \| Author \| Title \| Journal Name \| IF \| Publication Year \| # Authors \| Citations \| Country of Corresponding Author \| Source of Funding \| Conflict of Interest Statement \| Type of Intervention \| PRISMA Statement? \| PROSPERO? \| Meta-Analysis? \| \| --- \| --- \| --- \| --- \| --- \| --- \| --- \| --- \| --- \| --- \| --- \| --- \| --- \| --- \| \| Ray K. et al. (2000) \| Caregiver support for postpartum depression \| Cochrane database of systematic reviews \| 7.755 \| 2000 \| 2 \| 66 \| Canada \| Institution \| Yes \| Peer support, Psychological support, Group therapy, Home Visitation \| No \| No \| No \| \| Lawrie T. et al. (2000) \| Oestrogens and progestogens for preventing and treating postnatal depression. \| The Cochrane database of systematic reviews \| 7.7550 \| 2000 \| 3 \| 132 \| Canada \| Insitution \| Yes \| Hormone therapy, hormonal contrnception use \| no \| no \| No \| \| Hoffbrand S. et al. (2001) \| Antidepressant drug treatment for postnatal depression. \| The Cochrane database of systematic reviews \| 7.7550 \| 2001 \| 3 \| 136 \| England \| Institution \| Yes \| Antidepressant use \| No \| No \| No \| \| Lumley J. et al. (2004) \| Intervening to reduce depression after birth: A systematic review of the randomized trials \| International Journal of Technology Assessment in Health Care \| 1.3330 \| 2004 \| 3 \| 97 \| Australia \| Not reported \| No \| group therapy, psychological support, educational programs, other, home visitation, peer support, \| No \| No \| Yes \| \| Dennis CL. (2005) \| Psychosocial and psychological interventions for prevention of postnatal depression: Systematic review \| British Medical Journal \| 27.6040 \| 2005 \| 1 \| 462 \| Canada \| None \| Yes \| Psychological support, home visitation, educational programs \| No \| No \| yes \| \| Shaw E. et al.(2006) \| Systematic review of the literature on postpartum care: Effectiveness of postpartum support to improve maternal parenting, mental health, quality of life, and physical health \| Birth-Issues in Perinatal Care \| 2.129 \| 2006 \| 4 \| 294 \| Canada \| Government \| No \| Home visitation, Peer support, Educational program, Others \| No \| No \| No \| \| Leahy-Warren P et al. (2007) \| Postnatal Depression: Prevalence, Mothers' Perspectives, and Treatments \| Archives of Psychiatric Nursing \| 1.2990 \| 2007 \| 2 \| 159 \| Ireland \| not reported \| No \| Antidepressant use, psychological support, peer support, educational programs, CBT \| No \| no \| No \| \| Poobal.an AS. et al. (2007 \| Effects of treating postnatal depression on mother-infant interaction and child development: Systematic review \| British Journal of Psychiatry \| 7.233 \| 2007 \| 6 \| 251 \| UK \| Government \| Yes \| Psychological Support, CBT, Home visitation, Group therapy, others \| No \| No \| No \| \| Dennis, CL et al. (20017 \| Psychosocial and psychological interventions for treating postpartum depression \| The Cochrane database of systematic reviews \| 7.7550 \| 2007 \| 2 \| 487 \| Canada \| Institution \| Yes \| Psychological support, CBT, other, antidepressant therapy \| No \| No \| Yes \| \| Dal.e, J et al. (2008) \| Peer support telephone calls for improving health. \| Cochrane Database of Systematic Reviews \| N/A \| 2008 \| 4 \| 146 \| england \| Institution \| Yes \| peer support \| no \| no \| no \| \| Dennis CL et al. (2008) \| A systematic review of telephone support for women during pregnancy and the early postpartum period \| JOGNN - Journal of Obstetric, Gynecologic, and Neonatal Nursing \| 1.2610 \| 2008 \| 2 \| 124 \| Canada \| Not reported \| No \| Other \| No \| No \| No \| \| Grigoriadis, S et al. (2009) \| Traditional postpartum practices and rituals: Clinical implications \| Canadian Journal of Psychiatr \| 4.0800 \| 2009 \| 7 \| 33 \| Canada \| None \| No \| other, traditional \| No \| No \| No \| \| Leis JA. et al. (2009) \| A systematic review of home-based interventions to prevent and treat postpartum depression \| Archives of Women's Mental Health \| 2.3480 \| 2009 \| 4 \| 76 \| United States \| Government, Institution \| No \| CBT, educational programs, educational programs, other \| No \| No \| No \| \| Craig M. et al. (2009) \| Postnatal depression \| BMJ clinical evidence \| 1.06 \| 2009 \| 2 \| 32 \| england \| Not reported \| Yes \| antidepressant use, Hormonal contraception use, Educational Programs, Group Therapy, CBT, Peer Support, Physical Activity, Psychological Support, Other \| no \| no \| no \| \| Dal.ey A. et al. (2009) \| The effectiveness of exercise in the management of post-natal depression: Systematic review and meta-analysis \| Family Practice \| 1.52 \| 2009 \| 3 \| 98 \| england \| None \| Yes \| Physical activity \| no \| no \| yes \| \| Stevenson MD. et al. (2010) \| Group cognitive behavioural therapy for postnatal depression: A systematic review of clinical effectiveness, costeffectiveness and value of information analyses \| Health Technology Assessment \| 3.819 \| 2010 \| 8 \| 49 \| UK \| Not reported \| No \| CBT, Group therapy \| No \| No \| No \| \| Ng RC. et al. (2010) \| Pharmacologic treatment for postpartum depression: A systematic review \| Pharmacotherapy \| 3.045 \| 2010 \| 5 \| 38 \| USA \| Institution \| No \| Antidepressant use, Chinese/traditional (hebal), Hormone treatment, others \| No \| No \| No \| \| Goodman JH. et al. (2011) \| Group treatment for postpartum depression: A systematic review \| Archives of Women's Mental Health \| 2.3480 \| 2011 \| 2 \| 63 \| United States \| Not reported \| No \| group therapy, (CBT, peer support, educational programs, psychological support) \| No \| No \| No \| \| Ni PK. et al. (2011) \| The role of family and friends in providing social support towards enhancing the wellbeing of postpartum women: a comprehensive systematic review. \| JBI library of systematic reviews \| N/A \| 2011 \| 2 \| 9 \| Singapore \| Not reported \| Yes \| Peer support \| No \| No \| No \| \| Nscimento SL. et al. (2012) \| Physical exercise during pregnancy: A systematic review \| Current Opinion in Obstetrics and Gynecology \| 2.411 \| 2012 \| 3 \| 271 \| Brazil \| None \| Yes \| Physical Activity \| No \| No \| No \| \| Sado M. et al. (2012) \| Hypnosis during pregnancy, childbirth, and the postnatal period for preventing postnatal depression \| Cochrane Database of Systematic Reviews \| 7.755 \| 2012 \| 4 \| 18 \| Japan \| Institution \| Yes \| Others (hypnosis) \| No \| No \| No \| \| Scope A. et al. (2012) \| Women's perceptions and experiences of group cognitive behaviour therapy and other group interventions for postnatal depression: A qualitative synthesis \| Journal of Advanced Nursing \| 2.376 \| 2012 \| 3 \| 16 \| UK \| Government \| Yes \| CBT, Group Therapy \| No \| No \| No \| \| Dodd JM. et al. (2012) \| Specialised antenatal clinics for women with a multiple pregnancy for improving maternal and infant outcomes. \| The Cochrane database of systematic reviews \| 7.7550 \| 2012 \| 2 \| N/A \| Australia \| Not reported \| Yes \| Home visitation, other \| No \| No \| Yes \| \| Sharma V. et al. (2013) \| Are antidepressants effective in the treatment of postpartum depression? A systematic review \| Primary Care Companion to the Journal of Clinical Psychiatry \| N/A \| 2013 \| 2 \| 26 \| Canada \| None \| Yes \| CBT, Psychological Support \| No \| No \| No \| \| Perveen T. et al. (2013) \| Long term effectiveness of cognitive behavior therapy for treatment of postpartum depression: A systematic review and meta-analysis \| Journal of Pakistan Medical Students \| N/A \| 2013 \| 5 \| 3 \| Pakistan \| None \| Yes \| CBT \| Yes \| No \| Yes \| \| Rahman A. et al. (2013) \| Interventions for common perinatal mental disorders in women in low- and middle-income countries: A systematic review and meta-analysis \| Bulletin of the World Health Organization \| 6.818 \| 2013 \| 8 \| 220 \| UK \| Government \| Yes \| CBT, Educational Program, Group Therapy, Antidepressant, Home Visitation, Others \| No \| No \| Yes \| \| Miller BJ. et al. (2013) \| Dietary supplements for preventing postnatal depression \| The Cochrane database of systematic reviews \| 7.7550 \| 2013 \| 5 \| 77 \| australia \| Insitution \| Yes \| other \| No \| No \| Yes \| \| Scope A. et al. (2013) \| Is group cognitive behaviour therapy for postnatal depression evidence-based practice? A systematic review \| BMC Psychiatry \| 2.666 \| 2013 \| 7 \| 31 \| UK \| Not reported \| Yes \| CBT, Group Therapy, Educational program \| No \| No \| Yes \| \| Lavender T. et al. (2013) \| Telephone support for women during pregnancy and the first six weeks postpartum \| Cochrane Database of Systematic Reviews \| 7.7550 \| 2013 \| 5 \| 92 \| England \| Not reported \| Yes \| Other \| No \| No \| Yes \| \| Miniati M. et al. (2014) \| Interpersonal psychotherapy for postpartum depression: A systematic review \| Archives of Women's Mental Health \| 2.348 \| 2014 \| 7 \| 79 \| Italy \| Not reported \| Yes \| Psychological Support \| No \| No \| No \| \| McDonagh MS. et al. (2014) \| Depression drug treatment outcomes in pregnancy and the postpartum period: A systematic review and meta-analysis \| Obstetrics and Gynecology \| 4.9820 \| 2014 \| 7 \| 64 \| United States \| Institution, government \| Yes \| antidepressant use \| No \| No \| yes \| \| De Crescenzo F. et al. (2014) \| Selective serotonin reuptake inhibitors (SSRIs) for post-partum depression (PPD): A systematic review of randomized clinical trials \| Journal of Affective Disorders \| 4.0840 \| 2014 \| 4 \| 72 \| Italy \| None \| Yes \| Antidepressant use \| no \| no \| no \| \| Yonemoto N. et al. (2017) \| Schedules for home visits in the early postpartum period \| Cochrane database of systematic reviews \| 7.755 \| 2014 \| 4 \| 113 \| Japan \| Government \| Yes \| Home visitation \| No \| No \| Yes \| \| Molyneaux E. et al. (2014) \| Antidepressant treatment for postnatal depression \| Cochrane Database of Systematic Reviews \| 7.755 \| 2014 \| 5 \| 102 \| UK \| Government, Philanthropic, Industry \| Yes \| Antidepressant use, CBT, Peer support, others \| Yes \| No \| Yes \| \| Gressier F. et al. (2015) \| Postpartum electroconvulsive therapy: A systematic review and case report \| General Hospital Psychiatry \| 3.2200 \| 2015 \| 4 \| 17 \| France \| Not reported \| No \| Other \| No \| No \| No \| \| Perry M. et al. (2015) \| Community-based interventions for improving maternal health and for reducing maternal health inequalities in high-income countries: A systematic map of research \| Globalization and Health \| 2.554 \| 2015 \| 6 \| 5 \| UK \| Government \| Yes \| Home visitation, Peer support, others \| No \| No \| No \| \| Tsivos ZL. et al. (2015) \| Interventions for postnatal depression assessing the mother-infant relationship and child developmental outcomes: A systematic review \| International Journal of Women's Health \| N/A \| 2015 \| 4 \| 62 \| UK \| Not reported \| Yes \| Peer support, CBT, Psychological Support, Antidepressant use, others (dyadic interaction) \| No \| No \| No \| \| Dodd JM. et al. (2015) \| Specialised antenatal clinics for women with a multiple pregnancy for improving maternal and infant outcomes. \| The Cochrane database of systematic reviews \| 7.7550 \| 2015 \| 3 \| 40 \| Australia \| Institution \| Yes \| Home visitation, other \| no \| no \| Yes \| \| Gilinsky AS. et al. (2015) \| Efficacy of physical activity interventions in post-natal populations: systematic review, meta-analysis and content coding of behaviour change techniques \| Health psychology review \| 7.2410 \| 2015 \| 6 \| 33 \| Scotland \| Institution \| No \| Physical activity \| Yes \| No \| yes \| \| Yargawa J. et al. (2015) \| Male involvement and maternal health outcomes: systematic review and meta-analysis \| Journal of epidemiology and community health \| 3.872 \| 2015 \| 2 \| 160 \| UK \| Institution \| Yes \| Others (Male involvment) \| Yes \| No \| Yes \| \| Mah BL. (2016) \| Oxytocin, Postnatal Depression, and Parenting: A Systematic Review \| Harvard Review of Psychiatry \| 3.2640 \| 2016 \| 1 \| 26 \| Australia \| Not reported \| Yes \| hormonee treatment \| No \| No \| No \| \| Saccibe G. et al. (2016) \| Omega-3 long-chain polyunsaturated fatty acids and fish oil supplementation during pregnancy: Which evidence? \| Journal of Maternal-Fetal and Neonatal Medicine \| 1.569 \| 2016 \| 3 \| 71 \| USA \| None \| Yes \| Others (dietary) \| Yes \| Yes \| No \| \| Stephens S. et al. (2016) \| Effectiveness of Psychological Interventions for Postnatal Depression in Primary Care: A Meta-Analysis \| Annals of family medicine \| 4.185 \| 2016 \| 4 \| 33 \| UK \| Not reported \| Yes \| CBT, Psychological Support, Group therapy, others \| Yes \| No \| Yes \| \| Madden K. et al. (2016) \| Hypnosis for pain management during labour and childbirth. \| The Cochrane database of systematic reviews \| 7.7550 \| 2016 \| 5 \| 167 \| Australia \| Institution \| Yes \| other \| No \| No \| Yes \| \| O'Connor E. et al. (2016) \| Primary care screening for and treatment of depression in pregnant and postpartumwomen evidence report and systematic review for the US preventive services task force \| JAMA - Journal of the American Medical Association \| 51.273 \| 2016 \| 5 \| 264 \| USA \| Government \| Yes \| CBT, Antidepressant \| No \| No \| Yes \| \| Li Y. et al. (2016) \| Chinese Herbal Medicine for Postpartum Depression: A Systematic Review of Randomized Controlled Trials \| Evidence-based Complementary and Alternative Medicine \| 2.0640 \| 2016 \| 7 \| 5 \| China \| Institution \| Yes \| Chinese/traditional \| No \| Yes \| Yes \| \| Dixon S. et al. (2017) \| Best practice for community-based management of postnatal depression in developing countries: A systematic review \| Health care for women international \| 0.9500 \| 2017 \| 2 \| 4 \| Australia \| Not reported \| No \| Home visitation, CBT, group therapy, peer support, \| Yes \| No \| No \| \| Hadfield H. et al. (2017) \| Women's Experiences of Seeking and Receiving Psychological and Psychosocial Interventions for Postpartum Depression: A Systematic Review and Thematic Synthesis of the Qualitative Literature \| Journal of Midwifery and Women's Health \| 1.0480 \| 2017 \| 2 \| 24 \| England \| Not reported \| Yes \| Psychological support \| yes \| no \| No \| \| Hsaing H. et al. (2017) \| Collaborative Care for Women With Depression: A Systematic Review \| Psychosomatics \| 1.6600 \| 2017 \| 6 \| 13 \| United States \| Not reported \| No \| Psychological support \| Yes \| No \| No \| \| Sal.igheh M. et al. (2017) \| Can exercise or physical activity help improve postnatal depression and weight loss? A systematic review \| Archives of Women's Mental Health \| 2.348 \| 2017 \| 4 \| 10 \| Australia \| None \| Yes \| Physical Activity \| Yes \| No \| No \| \| Mendelson T. et al. (2017) \| NICU-based interventions to reduce maternal depressive and anxiety symptoms: A meta-analysis \| Pediatrics \| 5.4170 \| 2017 \| 5 \| 28 \| United States \| None \| Yes \| other \| No \| No \| Yes \| \| Suto M. et al. (2017) \| Effects of prenatal childbirth education for partners of pregnant women on paternal postnatal mental health and couple relationship: A systematic review \| Journal of Affective Disorders \| 4.084 \| 2017 \| 4 \| 21 \| Japan \| Institution \| Yes \| Educational program \| Yes \| Yes \| No \| \| Dhillon A. et al. (2017) \| Mindfulness-Based Interventions During Pregnancy: a Systematic Review and Meta-analysis. \| Mindfulness \| 3.0000 \| 2017 \| 3 \| 42 \| England \| None \| Yes \| Other \| Yes \| yes \| yes \| \| Pritchett RV. et al. (2017) \| Does aerobic exercise reduce postpartum depressive symptoms?: A systematic review and meta-analysis \| British Journal of General Practice \| 4.434 \| 2017 \| 3 \| 21 \| UK \| Government and Institution \| Yes \| Physical Activity \| Yes \| Yes \| Yes \| \| Sikorski C. et al. (2018) \| Could Postnatal Women's Groups Be Used to Improve Outcomes for Mothers and Children in High-Income Countries? A Systematic Review \| Maternal and child health journal \| 1.736 \| 2018 \| 7 \| 3 \| UK \| Government and Institution \| No \| Group Therapy \| Yes \| No \| No \| \| Sangsawang B. et al. (2019) \| Interventions for the prevention of postpartum depression in adolescent mothers: a systematic review \| Archives of Women's Mental Health \| 2.348 \| 2018 \| 3 \| 8 \| Thailand \| Not reported \| Yes \| Home visitation, Educational program, Peer support, CBT, Group therapy, others \| Yes \| No \| No \| \| Gurung B. et al. (2018) \| Identifying and assessing the benefits of interventions for postnatal depression: A systematic review of economic evaluations \| BMC Pregnancy and Childbirth \| 2.4130 \| 2018 \| 5 \| 7 \| England \| Not reported \| Yes \| peer support, CBT, antidepresasant therapy, group therapy, educational programs, home visitation, psychological support, other \| yes \| no \| No \| \| Nair U. et al. (2018) \| The effectiveness of telemedicine interventions to address maternal depression: A systematic review and meta-analysis \| Journal of telemedicine and telecare \| 2.229 \| 2018 \| 4 \| 5 \| Australia \| None \| Yes \| CBT, Online Therapy, Group Therapy, others \| Yes \| Yes \| Yes \| \| Sun Y. et al. (2018) \| Treatment of depression with Chai Hu Shu Gan San: A systematic review and meta-analysis of 42 randomized controlled trials \| BMC Complementary and Alternative Medicine \| 2.479 \| 2018 \| 4 \| 12 \| China \| None \| Yes \| Chinese/Traditional, Antidepressant use \| Yes \| No \| Yes \| \| Huang L. et al. (2018) \| Is cognitive behavioral therapy a better choice for women with postnatal depression? A systematic review and meta-analysis \| PLoS ONE \| 2.7760 \| 2018 \| 4 \| 8 \| China \| None \| Yes \| CBT \| Yes \| Yes \| Yes \| \| Davenport MH. et al. (2018) \| Impact of prenatal exercise on both prenatal and postnatal anxiety and depressive symptoms: a systematic review and meta-analysis \| British journal of sports medicine \| 11.65 \| 2018 \| 19 \| 30 \| Canada \| Institution \| Yes \| Physical activity \| YesY \| No \| Yes \| \| Molyneaux E. et al. (2018) \| Antidepressants for preventing postnatal depression \| Cochrane Database of Systematic Reviews \| 7.755 \| 2018 \| 6 \| 27 \| UK \| Institution \| Yes \| Antidepressant use, CBT, Peer support, others \| Yes \| No \| No \| \| Owais S. et al. (2018) \| Non-pharmacological interventions for improving postpartum maternal sleep: A systematic review and meta-analysis \| Sleep Medicine Reviews \| 10.517 \| 2018 \| 5 \| 9 \| Canada \| Not reported \| Yes \| Educational program, CBT, Psychological support, Chinese/traditional, Physical Activity, Others \| Yes \| Yes \| Yes \| \| Yang L. et al. (2018) \| A systematic review of acupuncture and Chinese herbal medicine for postpartum depression \| Complementary therapies in clinical practice \| 1.587 \| 2018 \| 8 \| 4 \| Australia \| Government and Institution \| Yes \| Chinese/Traditional, Antidepressant use \| No \| Yes \| Yes \| \| Li S. et al. (2018) \| Effectiveness of acupuncture in postpartum depression: a systematic review and meta-analysis \| Acupuncture in medicine : journal of the British Medical Acupuncture Society \| 2.2750 \| 2018 \| 4 \| N/A \| China \| Institution \| Yes \| Chinese/traditional \| Yes \| Yes \| Yes \| \| Middleton P. et al. (2018) \| Omega-3 fatty acid addition during pregnancy \| The Cochrane database of systematic reviews \| 7.7550 \| 2018 \| 6 \| 84 \| australia \| Institution, government \| Yes \| other \| No \| No \| Yes \| \| Kolomanska-Bogucka D. et al. (2019) \| Physical Activity and the Occurrence of Postnatal Depression-A Systematic Review \| Medicina (Kaunas, Lithuania) \| 1.5000 \| 2019 \| 2 \| 1 \| Poland \| None \| Yes \| Physical activity \| Yes \| No \| No \| \| Westerhoff B. et al. (2019) \| About:blank? Online Interventions for Postpartum Depression \| Verhaltenstherapie \| 1.073 \| 2019 \| 3 \| 1 \| Germany \| Not reported \| Yes \| Online therapy, CBT, psychological support, Educational program, peer support, others \| Yes \| No \| No \| \| De Cagna F. et al. (2019). \| The role of intranasal oxytocin in anxiety and depressive disorders: A systematic review of randomized controlled trials \| European Neuropsychopharmacology \| 4.4680 \| 2019 \| 8 \| 14 \| Italy \| Not reported \| No \| Hormone therapy \| Yes \| No \| No \| \| Rezaie-Keikhaie K. et al. (2019) \| Effect of aromatherapy on post-partum complications: A systematic review \| Complementary therapies in clinical practice \| 1.587 \| 2019 \| 8 \| 1 \| Iran \| None \| Yes \| Others (Aromatherapy) \| Yes \| No \| No \| \| Tong P. et al. (2019) \| Traditional Chinese acupuncture and postpartum depression: A systematic review and meta-analysis \| Journal of the Chinese Medical Association \| 1.894 \| 2019 \| 6 \| 1 \| China \| Government \| No \| Chinese/Traditional, Antidepressant use \| Yes \| No \| Yes \| \| Tsai SS. et al. (2019) \| The Effects of Aromatherapy on Postpartum Women: A Systematic Review \| Journal of nursing research \| 0.969 \| 2019 \| 3 \| N/A \| Taiwan \| Not reported \| Yes \| Others (Aromatherapy) \| Yes \| No \| No \| \| Li W. et al. (2019) \| Effectiveness of Acupuncture Used for the Management of Postpartum Depression: A Systematic Review and Meta-Analysis \| BioMed Research International \| 2.5830 \| 2019 \| 4 \| 11 \| China/USA \| Institution \| Yes \| Chinese/traditional \| Yes \| No \| Yes \| \| Scime NV. et al. (2019) \| The effect of skin-to-skin care on postpartum depression among mothers of preterm or low birthweight infants: A systematic review and meta-analysis \| Journal of Affective Disorders \| 4.084 \| 2019 \| 3 \| 2 \| Canada \| None \| Yes \| Others (skin to skin) \| Yes \| Yes \| Yes \| \| Ti A. et al. (2019) \| Postpartum hormonal contraception use and incidence of postpartum depression: a systematic review \| European Journal of Contraception and Reproductive Health Care \| 1.382 \| 2019 \| 2 \| 5 \| USA \| None \| Yes \| Hormonal Contraception use, Antidepressant use \| Yes \| No \| No \| \| Ganho-Avila A. et al. (2019) \| Efficacy of rTMS in decreasing postnatal depression symptoms: A systematic review \| Psychiatry Research \| 2.6820 \| 2019 \| 4 \| 2 \| Portugal \| Institution \| Yes \| Other \| Yes \| Yes \| No \| \| Nakamura A. et al. (2019) \| Physical activity during pregnancy and postpartum depression: Systematic review and meta-analysis \| Journal of Affective Disorders \| 4.084 \| 2019 \| 6 \| 8 \| France \| None \| Yes \| Physical Activity \| Yes \| Yes \| Yes \| \| Park S. et al. (2019) \| Effects of psychoeducation on the mental health and relationships of pregnant couples: A systemic review and meta-analysis \| International journal of nursing studies \| 3.57 \| 2019 \| 4 \| N/A \| Korea \| Government \| Yes \| Educational program \| No \| No \| Yes \| \| Yang WJ. et al. (2019) \| The effectiveness of music therapy for postpartum depression: A systematic review and meta-analysis \| Complementary therapies in clinical practice \| 1.587 \| 2019 \| 7 \| N/A \| China \| None \| Yes \| Music Therapy \| Yes \| Yes \| Yes \| \| Carter T. et al. (2019) \| The effectiveness of exercise-based interventions for preventing or treating postpartum depression: a systematic review and meta-analysis \| Archives of Women's Mental Health \| 2.35 \| 2019 \| 4 \| 7 \| england \| Institution \| Yes \| Physical activity \| yes \| yes \| yes \| \| Huang R. et al. (2020) \| The short- and long-term effectiveness of mother-infant psychotherapy on postpartum depression: A systematic review and meta-analysis \| Journal of Affective Disorders \| 4.0840 \| 2020 \| 7 \| 2 \| China \| Institution \| Yes \| Psychological support \| Yes \| No \| Yes \| \| Roman M. et al. (2020) \| The efficiency of online cognitive-behavioral therapy for postpartum depressive symptomatology: a systematic review and meta-analysis \| Women & health \| 1.162 \| 2020 \| 3 \| N/A \| Romania \| Not reported \| No \| Online therapy, CBT \| Yes \| No \| Yes \| \| Dol J,. et al. (2020) \| Impact of mobile health interventions during the perinatal period on maternal psychosocial outcomes: a systematic review \| JBI database of systematic reviews and implementation reports \| 0.7400 \| 2020 \| 6 \| 0 \| Canada \| Institution \| Yes \| Online therapy \| Yes \| No \| Yes \| |
| --- | --- | --- | --- | --- | --- | --- | --- | --- | --- | --- | --- | --- | --- | --- | --- | --- | --- | --- | --- | --- | --- | --- | --- | --- | --- | --- | --- | --- | --- | --- | --- | --- | --- | --- | --- | --- | --- | --- | --- | --- | --- | --- | --- | --- | --- | --- | --- | --- | --- | --- | --- | --- | --- | --- | --- | --- | --- | --- | --- | --- | --- | --- | --- | --- | --- | --- | --- | --- | --- | --- | --- | --- | --- | --- | --- | --- | --- | --- | --- | --- | --- | --- | --- | --- | --- | --- | --- | --- | --- | --- | --- | --- | --- | --- | --- | --- | --- | --- | --- | --- | --- | --- | --- | --- | --- | --- | --- | --- | --- | --- | --- | --- | --- | --- | --- | --- | --- | --- | --- | --- | --- | --- | --- | --- | --- | --- | --- | --- | --- | --- | --- | --- | --- | --- | --- | --- | --- | --- | --- | --- | --- | --- | --- | --- | --- | --- | --- | --- | --- | --- | --- | --- | --- | --- | --- | --- | --- | --- | --- | --- | --- | --- | --- | --- | --- | --- | --- | --- | --- | --- | --- | --- | --- | --- | --- | --- | --- | --- | --- | --- | --- | --- | --- | --- | --- | --- | --- | --- | --- | --- | --- | --- | --- | --- | --- | --- | --- | --- | --- | --- | --- | --- | --- | --- | --- | --- | --- | --- | --- | --- | --- | --- | --- | --- | --- | --- | --- | --- | --- | --- | --- | --- | --- | --- | --- | --- | --- | --- | --- | --- | --- | --- | --- | --- | --- | --- | --- | --- | --- | --- | --- | --- | --- | --- | --- | --- | --- | --- | --- | --- | --- | --- | --- | --- | --- | --- | --- | --- | --- | --- | --- | --- | --- | --- | --- | --- | --- | --- | --- | --- | --- | --- | --- | --- | --- | --- | --- | --- | --- | --- | --- | --- | --- | --- | --- | --- | --- | --- | --- | --- | --- | --- | --- | --- | --- | --- | --- | --- | --- | --- | --- | --- | --- | --- | --- | --- | --- | --- | --- | --- | --- | --- | --- | --- | --- | --- | --- | --- | --- | --- | --- | --- | --- | --- | --- | --- | --- | --- | --- | --- | --- | --- | --- | --- | --- | --- | --- | --- | --- | --- | --- | --- | --- | --- | --- | --- | --- | --- | --- | --- | --- | --- | --- | --- | --- | --- | --- | --- | --- | --- | --- | --- | --- | --- | --- | --- | --- | --- | --- | --- | --- | --- | --- | --- | --- | --- | --- | --- | --- | --- | --- | --- | --- | --- | --- | --- | --- | --- | --- | --- | --- | --- | --- | --- | --- | --- | --- | --- | --- | --- | --- | --- | --- | --- | --- | --- | --- | --- | --- | --- | --- | --- | --- | --- | --- | --- | --- | --- | --- | --- | --- | --- | --- | --- | --- | --- | --- | --- | --- | --- | --- | --- | --- | --- | --- | --- | --- | --- | --- | --- | --- | --- | --- | --- | --- | --- | --- | --- | --- | --- | --- | --- | --- | --- | --- | --- | --- | --- | --- | --- | --- | --- | --- | --- | --- | --- | --- | --- | --- | --- | --- | --- | --- | --- | --- | --- | --- | --- | --- | --- | --- | --- | --- | --- | --- | --- | --- | --- | --- | --- | --- | --- | --- | --- | --- | --- | --- | --- | --- | --- | --- | --- | --- | --- | --- | --- | --- | --- | --- | --- | --- | --- | --- | --- | --- | --- | --- | --- | --- | --- | --- | --- | --- | --- | --- | --- | --- | --- | --- | --- | --- | --- | --- | --- | --- | --- | --- | --- | --- | --- | --- | --- | --- | --- | --- | --- | --- | --- | --- | --- | --- | --- | --- | --- | --- | --- | --- | --- | --- | --- | --- | --- | --- | --- | --- | --- | --- | --- | --- | --- | --- | --- | --- | --- | --- | --- | --- | --- | --- | --- | --- | --- | --- | --- | --- | --- | --- | --- | --- | --- | --- | --- | --- | --- | --- | --- | --- | --- | --- | --- | --- | --- | --- | --- | --- | --- | --- | --- | --- | --- | --- | --- | --- | --- | --- | --- | --- | --- | --- | --- | --- | --- | --- | --- | --- | --- | --- | --- | --- | --- | --- | --- | --- | --- | --- | --- | --- | --- | --- | --- | --- | --- | --- | --- | --- | --- | --- | --- | --- | --- | --- | --- | --- | --- | --- | --- | --- | --- | --- | --- | --- | --- | --- | --- | --- | --- | --- | --- | --- | --- | --- | --- | --- | --- | --- | --- | --- | --- | --- | --- | --- | --- | --- | --- | --- | --- | --- | --- | --- | --- | --- | --- | --- | --- | --- | --- | --- | --- | --- | --- | --- | --- | --- | --- | --- | --- | --- | --- | --- | --- | --- | --- | --- | --- | --- | --- | --- | --- | --- | --- | --- | --- | --- | --- | --- | --- | --- | --- | --- | --- | --- | --- | --- | --- | --- | --- | --- | --- | --- | --- | --- | --- | --- | --- | --- | --- | --- | --- | --- | --- | --- | --- | --- | --- | --- | --- | --- | --- | --- | --- | --- | --- | --- | --- | --- | --- | --- | --- | --- | --- | --- | --- | --- | --- | --- | --- | --- | --- | --- | --- | --- | --- | --- | --- | --- | --- | --- | --- | --- | --- | --- | --- | --- | --- | --- | --- | --- | --- | --- | --- | --- | --- | --- | --- | --- | --- | --- | --- | --- | --- | --- | --- | --- | --- | --- | --- | --- | --- | --- | --- | --- | --- | --- | --- | --- | --- | --- | --- | --- | --- | --- | --- | --- | --- | --- | --- | --- | --- | --- | --- | --- | --- | --- | --- | --- | --- | --- | --- | --- | --- | --- | --- | --- | --- | --- | --- | --- | --- | --- | --- | --- | --- | --- | --- | --- | --- | --- | --- | --- | --- | --- | --- | --- | --- | --- | --- | --- | --- | --- | --- | --- | --- | --- | --- | --- | --- | --- | --- | --- | --- | --- | --- | --- | --- | --- | --- | --- | --- | --- | --- | --- | --- | --- | --- | --- | --- | --- | --- | --- | --- | --- | --- | --- | --- | --- | --- | --- | --- | --- | --- | --- | --- | --- | --- | --- | --- | --- | --- | --- | --- | --- | --- | --- | --- | --- | --- | --- | --- | --- | --- | --- | --- | --- | --- | --- | --- | --- | --- | --- | --- | --- | --- | --- | --- | --- | --- | --- | --- | --- | --- | --- | --- | --- | --- | --- | --- | --- | --- | --- | --- | --- | --- | --- | --- | --- | --- | --- | --- | --- | --- | --- | --- | --- | --- | --- | --- | --- | --- | --- | --- | --- | --- | --- | --- | --- | --- | --- | --- | --- | --- | --- | --- | --- | --- | --- | --- | --- | --- | --- | --- | --- | --- | --- | --- | --- | --- | --- | --- | --- | --- | --- | --- | --- | --- | --- | --- | --- | --- | --- | --- | --- | --- | --- | --- | --- | --- | --- | --- | --- | --- | --- | --- | --- | --- | --- | --- | --- | --- | --- | --- | --- | --- | --- | --- | --- | --- | --- | --- | --- | --- | --- | --- | --- | --- | --- | --- | --- | --- | --- | --- | --- | --- | --- | --- | --- | --- | --- | --- | --- | --- | --- | --- | --- | --- | --- | --- | --- | --- | --- | --- | --- | --- | --- | --- | --- | --- | --- | --- | --- | --- | --- | --- | --- | --- | --- | --- | --- | --- | --- | --- | --- | --- | --- | --- | --- | --- | --- | --- | --- | --- | --- | --- | --- | --- | --- | --- | --- | --- | --- | --- | --- | --- | --- | --- | --- | --- | --- | --- | --- | --- | --- | --- | --- | --- | --- | --- | --- | --- | --- | --- | --- | --- | --- | --- | --- | --- | --- | --- | --- | --- | --- | --- | --- | --- | --- | --- | --- | --- | --- | --- | --- | --- | --- | --- | --- | --- |

Table S2: List of Excluded Studies and Their Reasons

| \| Authors \| Title \| Published Year \| Journal \| Exclusion Reason: \| \| --- \| --- \| --- \| --- \| --- \| \| Sockol L.E. (2015) \| A systematic review of the efficacy of cognitive behavioral therapy for treating and preventing perinatal depression \| 2015 \| Journal of Affective Disorders \| Did not examine the effects of an intervention for treating PPD \| \| Smith CA et al. (2019) \| The effect of complementary medicines and therapies on maternal anxiety and depression in pregnancy: A systematic review and meta-analysis \| 2019 \| Journal of Affective Disorders \| Did not examine the effects of an intervention for treating PPD \| \| Scope A et al. (2017) \| Perceptions and experiences of interventions to prevent postnatal depression. A systematic review and qualitative evidence synthesis \| 2017 \| Journal of Affective Disorders \| Did not examine the effects of an intervention for treating PPD \| \| Rayce SB. et al. (2020) \| Effects of parenting interventions for mothers with depressive symptoms and an infant: systematic review and meta-analysis. \| 2020 \| BJPsych open \| Did not examine the effects of an intervention for treating PPD \| \| Poleszczyk A et al. (2019) \| State of the art and future perspectives on the use of non-invasive neuromodulation in peripartum psychiatric disorders \| 2019 \| Encephale \| Did not examine the effects of an intervention for treating PPD; \| \| Lee E. et al. (2015) \| Is there a role for a web-based mental health and wellbeing self-management resource for use during pregnancy? A systematic review \| 2015 \| Psychoneuroendocrinology \| Not a systematic review; \| \| Homer CS et al. (2012) \| Group versus conventional antenatal care for women. \| 2012 \| The Cochrane database of systematic reviews \| Did not examine the effects of an intervention for treating PPD \| \| Goodman J.H. (2011) \| Group treatment of postpartum depression: A systematic review \| 2011 \| Archives of Women's Mental Health \| Not a systematic review \| \| Dennis CL et al. (2013) \| Psychosocial and psychological interventions for preventing postpartum depression \| 2013 \| Cochrane Database of Systematic Reviews \| Did not examine the effects of an intervention for treating PPD \| \| Daley AJ et al. (2015) \| The effectiveness of exercise for the prevention and treatment of antenatal depression: Systematic review with meta-analysis \| 2015 \| BJOG: An International Journal of Obstetrics and Gynaecology \| Did not examine the effects of an intervention for treating PPD \| \| Christesen HT et al. (2012) \| The impact of vitamin D on pregnancy: A systematic review \| 2012 \| Acta Obstetricia et Gynecologica Scandinavica \| Did not examine the effects of an intervention for treating PPD \| \| Barlow J. et al. (2015) \| Parent-infant psychotherapy for improving parental and infant mental health. \| 2015 \| The Cochrane database of systematic reviews \| Did not examine the effects of an intervention for treating PPD \| \| Mehnert A. et al (2018) \| Global Health Challenges to Community-Based and Individual Psychosocial Intervention Strategies \| 2018 \| PPmP Psychotherapie Psychosomatik Medizinische Psychologie \| Did not examine the effects of an intervention for treating PPD \| \| Akioyamen LE. et al. (2016) \| Effects of depression pharmacotherapy in fertility treatment on conception, birth, and neonatal health: A systematic review \| 2016 \| Journal of Psychosomatic Research \| Did not examine the effects of an intervention for treating PPD \| \| Newberry SJ. et al. (2016) \| Omega-3 Fatty Acids and Maternal and Child Health: An Updated Systematic Review. \| 2016 \| Evidence report/technology assessment \| Did not examine the effects of an intervention for treating PPD \| \| Taylor BL. et al. (2016) \| The effectiveness of mindfulness-based interventions in the perinatal period: A systematic review and meta-analysis \| 2016 \| PLoS ONE \| Did not examine the effects of an intervention for treating PPD \| \| Ortega RM et al. (2012) \| Effects of omega 3 fatty acids supplementation in behavior and non-neurodegenerative neuropsychiatric disorders. \| 2012 \| The British journal of nutrition \| Did not examine the effects of an intervention for treating PPD \| \| Morrell CJ. et al. (2015) \| The prevention of postnatal depression: Anhta evidence synthesis following a systematic review of quantitative studies \| 2015 \| Archives of Women's Mental Health \| Did not examine the effects of an intervention for treating PPD \| \| Mori E et al. (2019) \| Effectiveness of parenting education for expectant primiparous women in Asian countries: A quantitative systematic review protocol \| 2019 \| JBI Database of Systematic Reviews and Implementation Reports \| Not a systematic review \| \| Sockol LE. et al. (2011) \| A meta-analysis of treatments for perinatal depression. \| 2011 \| Clinical psychology review \| Did not examine the effects of an intervention for treating PPD \| \| Olhaberry M. et al. (2013) \| Psychological perinatal interventions in maternal depression and mother-child bond: A systematic review \| 2013 \| Terapia Psicologica \| Did not examine the effects of an intervention for treating PPD \| \| O'Connor E et al. (2019) \| Interventions to Prevent Perinatal Depression: Evidence Report and Systematic Review for the US Preventive Services Task Force \| 2019 \| JAMA - Journal of the American Medical Association \| Did not examine the effects of an intervention for treating PPD \| \| Nillni YI. et al. (2018) \| Treatment of depression, anxiety, and trauma-related disorders during the perinatal period: A systematic review \| 2018 \| Clinical Psychology Review \| Did not examine the effects of an intervention for treating PPD \| \| Moore S. et al. (2018) \| A Systematic Review of Integrated Care Interventions Addressing Perinatal Depression Care in Ambulatory Obstetric Care Settings. \| 2018 \| Clinical obstetrics and gynecology \| Did not examine the effects of an intervention for treating PPD \| \| Loughnan SA. et al. (2019) \| Internet-delivered psychological interventions for clinical anxiety and depression in perinatal women: a systematic review and meta-analysis \| 2019 \| Archives of Women's Mental Health \| Did not examine the effects of an intervention for treating PPD \| \| Lin PY. et al. (2017) \| Polyunsaturated Fatty Acids in Perinatal Depression: A Systematic Review and Meta-analysis \| 2017 \| Biological Psychiatry \| Did not examine the effects of an intervention for treating PPD \| \| Lieberman K. et al. (2013) \| Systematic review of perinatal depression interventions for teen mothers \| 2013 \| Archives of Women's Mental Health \| Did not examine the effects of an intervention for treating PPD \| \| Lee EW. et al. (2016) \| Web-based interventions for prevention and treatment of perinatal mood disorders: A systematic review \| 2016 \| BMC Pregnancy and Childbirth \| Did not examine the effects of an intervention for treating PPD \| \| Hall H. et al. (2015) \| The effectiveness of mindfulness training on perinatal mental health; a systematic review \| 2015 \| Integrative Medicine Research \| Did not examine the effects of an intervention for treating PPD \| \| Gong H. et al. (2015) \| Yoga for prenatal depression: A systematic review and meta-analysis \| 2015 \| BMC Psychiatry \| Did not examine the effects of an intervention for treating PPD \| \| Gajaria A. et al. (2018) \| Interventions for perinatal depression in low and middle-income countries: A systematic review \| 2018 \| Asian Journal of Psychiatry \| Did not examine the effects of an intervention for treating PPD \| \| Fassaie S. et al. (2020) \| Maternal distress, HPA activity, and antenatal interventions: A systematic review \| 2020 \| Psychoneuroendocrinology \| Did not examine the effects of an intervention for treating PPD \| \| Dennis CL. et al. (2013) \| Interventions (other than pharmacological, psychosocial or psychological) for treating antenatal depression. \| 2013 \| The Cochrane database of systematic reviews \| Did not examine the effects of an intervention for treating PPD \| \| Dennis CL. et al. (2008) \| Interventions (other than pharmacological, psychosocial or psychological) for treating antenatal depression. \| 2008 \| The Cochrane database of systematic reviews \| Did not examine the effects of an intervention for treating PPD \| \| Dennis CL. (2014) \| Psychosocial interventions for the treatment of perinatal depression. \| 2014 \| Best practice & research. Clinical obstetrics & gynaecology \| Did not examine the effects of an intervention for treating PPD \| \| Dennis CL et al. (2007) \| Psychosocial and psychological interventions for treating antenatal depression. \| 2007 \| The Cochrane database of systematic reviews \| Did not examine the effects of an intervention for treating PPD \| \| Cluxton-Keller F. et al. (2018) \| Clinical effectiveness of family therapeutic interventions in the prevention and treatment of perinatal depression: A systematic review and meta-analysis \| 2018 \| PLoS ONE \| Did not examine the effects of an intervention for treating PPD \| \| Chowdhary N. et al. (2014) \| The content and delivery of psychological interventions for perinatal depression by non-specialist health workers in low and middle income countries: A systematic review \| 2014 \| Best Practice and Research: Clinical Obstetrics and Gynaecology \| Did not examine the effects of an intervention for treating PPD \| \| Chatillon O. et al. (2010) \| Antepartum depression: Prevalence, diagnosis and treatment \| 2010 \| Encephale \| Did not examine the effects of an intervention for treating PPD \| \| Catling CJ et al. (2015) \| Group versus conventional antenatal care for women. \| 2015 \| The Cochrane database of systematic reviews \| Did not examine the effects of an intervention for treating PPD \| \| Camacho EM et al. (2018) \| Cost-effectiveness of interventions for perinatal anxiety and/or depression: A systematic review \| 2018 \| BMJ Open \| Did not examine the effects of an intervention for treating PPD \| \| Byatt N. et al. (2015) \| Enhancing Participation in Depression Care in Outpatient Perinatal Care Settings: A Systematic Review \| 2015 \| Obstetrics and Gynecology \| Did not examine the effects of an intervention for treating PPD \| \| Ashford MT. et al. (2016) \| Computer- or web-based interventions for perinatal mental health: A systematic review \| 2016 \| Journal of Affective Disorders \| Did not examine the effects of an intervention for treating PPD \| \| Amini SJ. et al. (2019) \| Postpartum depression and vitamin D: A systematic review. \| 2019 \| Critical reviews in food science and nutrition \| Did not examine the effects of an intervention for treating PPD \| |
| --- | --- | --- | --- | --- | --- | --- | --- | --- | --- | --- | --- | --- | --- | --- | --- | --- | --- | --- | --- | --- | --- | --- | --- | --- | --- | --- | --- | --- | --- | --- | --- | --- | --- | --- | --- | --- | --- | --- | --- | --- | --- | --- | --- | --- | --- | --- | --- | --- | --- | --- | --- | --- | --- | --- | --- | --- | --- | --- | --- | --- | --- | --- | --- | --- | --- | --- | --- | --- | --- | --- | --- | --- | --- | --- | --- | --- | --- | --- | --- | --- | --- | --- | --- | --- | --- | --- | --- | --- | --- | --- | --- | --- | --- | --- | --- | --- | --- | --- | --- | --- | --- | --- | --- | --- | --- | --- | --- | --- | --- | --- | --- | --- | --- | --- | --- | --- | --- | --- | --- | --- | --- | --- | --- | --- | --- | --- | --- | --- | --- | --- | --- | --- | --- | --- | --- | --- | --- | --- | --- | --- | --- | --- | --- | --- | --- | --- | --- | --- | --- | --- | --- | --- | --- | --- | --- | --- | --- | --- | --- | --- | --- | --- | --- | --- | --- | --- | --- | --- | --- | --- | --- | --- | --- | --- | --- | --- | --- | --- | --- | --- | --- | --- | --- | --- | --- | --- | --- | --- | --- | --- | --- | --- | --- | --- | --- | --- | --- | --- | --- | --- | --- | --- | --- | --- | --- | --- | --- | --- | --- | --- | --- | --- | --- | --- | --- | --- | --- | --- | --- | --- | --- | --- | --- | --- | --- |

Table S3: AMSTAR Scoring of Included Studies

| \| Author \| 1. Was an 'a priori' design provided? \| 2. Was there duplicate study selection and data extraction? \| 3. Was a comprehensive literature search performed? \| 4. Was the status of publication used as an inclusion criterion? \| 5. Was a list of studies provided? \| 6. Were the characteristics of the included studies provided? \| 7. Was the scientific quality of the included studies assessed and documented \| 8. Was the scientific quality of the included studies used appropriately in formulating conclusions? \| 9. Were the methods used to combine the findings of studies appropriate? \| 10. Was the likelihood of publication bias assessed? \| 11. Was the conflict of interest included? \| Total \| \| --- \| --- \| --- \| --- \| --- \| --- \| --- \| --- \| --- \| --- \| --- \| --- \| --- \| \| Ray K. et al. (2000) \| N \| N \| Y \| N \| N \| Y \| Y \| N \| N \| N \| N \| 3 \| \| Lawrie T. et al. (2000) \| N \| Y \| Y \| Y \| Y \| Y \| Y \| Y \| N \| N \| N \| 7 \| \| Hoffbrand S. et al. (2001) \| N \| Y \| Y \| Y \| Y \| Y \| Y \| Y \| N \| N \| N \| 7 \| \| Lumley J. et al. (2004) \| N \| N \| Y \| Y \| N \| N \| N \| Y \| Y \| N \| N \| 4 \| \| Dennis CL. (2005) \| N \| N \| Y \| Y \| Y \| Y \| N \| Y \| Y \| N \| N \| 6 \| \| Shaw E. et al.(2006) \| N \| N \| Y \| N \| N \| Y \| Y \| N \| N \| N \| N \| 3 \| \| Leahy-Warren P et al. (2007) \| N \| N \| N \| N \| N \| Y \| N \| N \| N \| N \| N \| 1 \| \| Poobal.an AS. et al. (2007 \| N \| Y \| Y \| N \| N \| Y \| Y \| Y \| Y \| N \| N \| 6 \| \| Dennis, CL et al. (20017 \| Y \| Y \| Y \| N \| Y \| Y \| Y \| Y \| Y \| Y \| N \| 9 \| \| Dal.e, J et al. (2008) \| N \| Y \| Y \| N \| Y \| Y \| Y \| Y \| N \| N \| N \| 6 \| \| Dennis CL et al. (2008) \| N \| N \| Y \| Y \| Y \| Y \| Y \| Y \| N \| N \| N \| 6 \| \| Grigoriadis, S et al. (2009) \| N \| N \| Y \| N \| N \| N \| N \| N \| N \| N \| N \| 1 \| \| Leis JA. et al. (2009) \| N \| N \| Y \| N \| Y \| Y \| N \| N \| N \| N \| N \| 3 \| \| Craig M. et al. (2009) \| N \| N \| N \| Y \| N \| Y \| Y \| Y \| N \| N \| N \| 5 \| \| Dal.ey A. et al. (2009) \| N \| N \| Y \| N \| N \| Y \| Y \| Y \| Y \| Y \| N \| 6 \| \| Stevenson MD. et al. (2010) \| N \| N \| Y \| N \| N \| N \| N \| N \| N \| N \| N \| 1 \| \| Ng RC. et al. (2010) \| Y \| N \| Y \| N \| Y \| Y \| N \| N \| Y \| N \| N \| 5 \| \| Goodman JH. et al. (2011) \| N \| N \| Y \| Y \| N \| Y \| Y \| Y \| N \| N \| N \| 5 \| \| Ni PK. et al. (2011) \| N \| N \| Y \| Y \| Y \| Y \| Y \| N \| Y \| N \| N \| 6 \| \| Nscimento SL. et al. (2012) \| N \| Y \| Y \| N \| N \| Y \| N \| N \| N \| N \| N \| 3 \| \| Sado M. et al. (2012) \| N \| N \| Y \| Y \| Y \| Y \| Y \| Y \| N \| N \| N \| 6 \| \| Scope A. et al. (2012) \| Y \| N \| Y \| Y \| N \| Y \| Y \| Y \| N \| N \| N \| 6 \| \| Dodd JM. et al. (2012) \| N \| Y \| Y \| Y \| N \| Y \| Y \| Y \| N \| N \| N \| 6 \| \| Sharma V. et al. (2013) \| N \| N \| Y \| N \| N \| Y \| N \| N \| N \| N \| N \| 2 \| \| Perveen T. et al. (2013) \| N \| N \| Y \| N \| Y \| Y \| Y \| N \| Y \| N \| N \| 5 \| \| Rahman A. et al. (2013) \| N \| N \| Y \| Y \| N \| Y \| N \| N \| Y \| Y \| N \| 5 \| \| Miller BJ. et al. (2013) \| N \| Y \| Y \| Y \| Y \| Y \| Y \| Y \| N \| N \| N \| 7 \| \| Scope A. et al. (2013) \| Y \| N \| Y \| Y \| Y \| Y \| Y \| Y \| Y \| N \| N \| 8 \| \| Lavender T. et al. (2013) \| N \| Y \| Y \| Y \| Y \| Y \| Y \| Y \| Y \| N \| N \| 8 \| \| Miniati M. et al. (2014) \| N \| N \| Y \| N \| N \| Y \| N \| N \| N \| N \| N \| 2 \| \| McDonagh MS. et al. (2014) \| N \| N \| Y \| N \| N \| Y \| N \| N \| Y \| N \| N \| 3 \| \| De Crescenzo F. et al. (2014) \| N \| y \| Y \| Y \| Y \| Y \| Y \| Y \| N \| N \| N \| 7 \| \| Yonemoto N. et al. (2017) \| N \| N \| Y \| Y \| Y \| Y \| Y \| Y \| Y \| Y \| N \| 8 \| \| Molyneaux E. et al. (2014) \| N \| Y \| Y \| Y \| Y \| Y \| Y \| Y \| Y \| Y \| Y \| 10 \| \| Gressier F. et al. (2015) \| N \| N \| Y \| N \| N \| Y \| N \| N \| N \| N \| N \| 2 \| \| Perry M. et al. (2015) \| Y \| N \| Y \| N \| N \| Y \| N \| N \| N \| N \| Y \| 4 \| \| Tsivos ZL. et al. (2015) \| N \| N \| Y \| N \| N \| Y \| Y \| Y \| N \| N \| N \| 4 \| \| Dodd JM. et al. (2015) \| N \| Y \| Y \| N \| N \| Y \| Y \| Y \| N \| N \| N \| 5 \| \| Gilinsky AS. et al. (2015) \| N \| N \| Y \| N \| N \| Y \| Y \| Y \| Y \| Y \| N \| 6 \| \| Yargawa J. et al. (2015) \| Y \| Y \| Y \| Y \| Y \| Y \| Y \| Y \| Y \| Y \| N \| 10 \| \| Mah BL. (2016) \| N \| N \| N \| N \| N \| Y \| N \| N \| N \| N \| N \| 1 \| \| Saccibe G. et al. (2016) \| Y \| Y \| Y \| N \| N \| Y \| Y \| N \| N \| N \| N \| 5 \| \| Stephens S. et al. (2016) \| N \| N \| Y \| N \| N \| Y \| Y \| Y \| Y \| Y \| N \| 6 \| \| Madden K. et al. (2016) \| N \| Y \| Y \| Y \| Y \| Y \| Y \| Y \| N \| N \| N \| 7 \| \| O'Connor E. et al. (2016) \| Y \| Y \| Y \| N \| N \| Y \| Y \| Y \| Y \| Y \| N \| 8 \| \| Li Y. et al. (2016) \| Y \| Y \| Y \| N \| N \| Y \| Y \| Y \| Y \| Y \| N \| 8 \| \| Dixon S. et al. (2017) \| N \| N \| N \| N \| N \| Y \| Y \| Y \| N \| N \| N \| 3 \| \| Hadfield H. et al. (2017) \| N \| N \| Y \| N \| N \| Y \| Y \| N \| N \| N \| N \| 3 \| \| Hsaing H. et al. (2017) \| N \| N \| Y \| Y \| N \| Y \| N \| Y \| N \| N \| N \| 4 \| \| Sal.igheh M. et al. (2017) \| N \| N \| Y \| Y \| N \| Y \| Y \| Y \| N \| N \| N \| 5 \| \| Mendelson T. et al. (2017) \| N \| N \| Y \| N \| N \| Y \| Y \| Y \| Y \| Y \| N \| 6 \| \| Suto M. et al. (2017) \| Y \| N \| Y \| N \| Y \| Y \| Y \| Y \| Y \| N \| N \| 7 \| \| Dhillon A. et al. (2017) \| Y \| Y \| Y \| Y \| Y \| Y \| N \| N \| Y \| N \| N \| 7 \| \| Pritchett RV. et al. (2017) \| Y \| Y \| Y \| Y \| N \| Y \| Y \| Y \| Y \| Y \| N \| 9 \| \| Sikorski C. et al. (2018) \| N \| N \| Y \| N \| N \| Y \| N \| N \| N \| N \| N \| 2 \| \| Sangsawang B. et al. (2019) \| N \| N \| Y \| N \| N \| Y \| Y \| Y \| N \| N \| N \| 4 \| \| Gurung B. et al. (2018) \| N \| N \| Y \| Y \| N \| Y \| Y \| N \| N \| N \| N \| 4 \| \| Nair U. et al. (2018) \| Y \| N \| Y \| N \| N \| Y \| Y \| Y \| Y \| N \| N \| 6 \| \| Sun Y. et al. (2018) \| N \| Y \| Y \| N \| N \| Y \| Y \| Y \| Y \| N \| N \| 6 \| \| Huang L. et al. (2018) \| N \| N \| Y \| N \| N \| Y \| Y \| Y \| Y \| Y \| N \| 6 \| \| Davenport MH. et al. (2018) \| N \| N \| Y \| N \| Y \| Y \| Y \| Y \| Y \| Y \| N \| 7 \| \| Molyneaux E. et al. (2018) \| N \| Y \| Y \| Y \| Y \| Y \| Y \| Y \| N \| N \| Y \| 8 \| \| Owais S. et al. (2018) \| Y \| N \| Y \| N \| Y \| Y \| Y \| Y \| Y \| Y \| N \| 8 \| \| Yang L. et al. (2018) \| Y \| N \| Y \| Y \| N \| Y \| Y \| Y \| Y \| Y \| N \| 8 \| \| Li S. et al. (2018) \| Y \| Y \| Y \| Y \| N \| Y \| Y \| Y \| Y \| N \| N \| 8 \| \| Middleton P. et al. (2018) \| N \| Y \| Y \| Y \| Y \| Y \| Y \| Y \| Y \| Y \| N \| 9 \| \| Kolomanska-Bogucka D. et al. (2019) \| N \| N \| N \| N \| N \| Y \| N \| N \| N \| N \| N \| 1 \| \| Westerhoff B. et al. (2019) \| N \| N \| Y \| N \| N \| Y \| N \| N \| N \| N \| N \| 2 \| \| De Cagna F. et al. (2019). \| N \| N \| Y \| N \| N \| Y \| Y \| N \| N \| N \| N \| 3 \| \| Rezaie-Keikhaie K. et al. (2019) \| Y \| N \| Y \| N \| N \| Y \| Y \| N \| Y \| N \| N \| 5 \| \| Tong P. et al. (2019) \| N \| N \| Y \| N \| N \| Y \| Y \| N \| Y \| Y \| N \| 5 \| \| Tsai SS. et al. (2019) \| N \| Y \| Y \| N \| N \| Y \| Y \| Y \| N \| N \| N \| 5 \| \| Li W. et al. (2019) \| N \| Y \| N \| N \| N \| Y \| Y \| Y \| Y \| Y \| N \| 6 \| \| Scime NV. et al. (2019) \| Y \| Y \| Y \| N \| N \| Y \| Y \| Y \| Y \| N \| N \| 7 \| \| Ti A. et al. (2019) \| Y \| Y \| Y \| N \| N \| Y \| Y \| Y \| Y \| N \| N \| 7 \| \| Ganho-Avila A. et al. (2019) \| Y \| Y \| Y \| Y \| N \| Y \| Y \| Y \| N \| N \| N \| 7 \| \| Nakamura A. et al. (2019) \| Y \| Y \| Y \| N \| N \| Y \| Y \| Y \| Y \| Y \| N \| 8 \| \| Park S. et al. (2019) \| N \| Y \| Y \| Y \| N \| Y \| Y \| Y \| Y \| Y \| N \| 8 \| \| Yang WJ. et al. (2019) \| Y \| Y \| Y \| N \| N \| Y \| Y \| Y \| Y \| Y \| N \| 8 \| \| Carter T. et al. (2019) \| Y \| Y \| Y \| Y \| n \| Y \| Y \| Y \| Y \| Y \| N \| 9 \| \| Huang R. et al. (2020) \| N \| N \| Y \| N \| N \| N \| Y \| Y \| Y \| N \| N \| 4 \| \| Roman M. et al. (2020) \| N \| N \| Y \| Y \| N \| Y \| N \| N \| Y \| Y \| N \| 5 \| \| Dol J,. et al. (2020) \| Y \| Y \| Y \| Y \| Y \| Y \| Y \| Y \| Y \| N \| N \| 9 \| \|  \|  \|  \|  \|  \|  \|  \|  \|  \|  \|  \|  \|  \| |
| --- | --- | --- | --- | --- | --- | --- | --- | --- | --- | --- | --- | --- | --- | --- | --- | --- | --- | --- | --- | --- | --- | --- | --- | --- | --- | --- | --- | --- | --- | --- | --- | --- | --- | --- | --- | --- | --- | --- | --- | --- | --- | --- | --- | --- | --- | --- | --- | --- | --- | --- | --- | --- | --- | --- | --- | --- | --- | --- | --- | --- | --- | --- | --- | --- | --- | --- | --- | --- | --- | --- | --- | --- | --- | --- | --- | --- | --- | --- | --- | --- | --- | --- | --- | --- | --- | --- | --- | --- | --- | --- | --- | --- | --- | --- | --- | --- | --- | --- | --- | --- | --- | --- | --- | --- | --- | --- | --- | --- | --- | --- | --- | --- | --- | --- | --- | --- | --- | --- | --- | --- | --- | --- | --- | --- | --- | --- | --- | --- | --- | --- | --- | --- | --- | --- | --- | --- | --- | --- | --- | --- | --- | --- | --- | --- | --- | --- | --- | --- | --- | --- | --- | --- | --- | --- | --- | --- | --- | --- | --- | --- | --- | --- | --- | --- | --- | --- | --- | --- | --- | --- | --- | --- | --- | --- | --- | --- | --- | --- | --- | --- | --- | --- | --- | --- | --- | --- | --- | --- | --- | --- | --- | --- | --- | --- | --- | --- | --- | --- | --- | --- | --- | --- | --- | --- | --- | --- | --- | --- | --- | --- | --- | --- | --- | --- | --- | --- | --- | --- | --- | --- | --- | --- | --- | --- | --- | --- | --- | --- | --- | --- | --- | --- | --- | --- | --- | --- | --- | --- | --- | --- | --- | --- | --- | --- | --- | --- | --- | --- | --- | --- | --- | --- | --- | --- | --- | --- | --- | --- | --- | --- | --- | --- | --- | --- | --- | --- | --- | --- | --- | --- | --- | --- | --- | --- | --- | --- | --- | --- | --- | --- | --- | --- | --- | --- | --- | --- | --- | --- | --- | --- | --- | --- | --- | --- | --- | --- | --- | --- | --- | --- | --- | --- | --- | --- | --- | --- | --- | --- | --- | --- | --- | --- | --- | --- | --- | --- | --- | --- | --- | --- | --- | --- | --- | --- | --- | --- | --- | --- | --- | --- | --- | --- | --- | --- | --- | --- | --- | --- | --- | --- | --- | --- | --- | --- | --- | --- | --- | --- | --- | --- | --- | --- | --- | --- | --- | --- | --- | --- | --- | --- | --- | --- | --- | --- | --- | --- | --- | --- | --- | --- | --- | --- | --- | --- | --- | --- | --- | --- | --- | --- | --- | --- | --- | --- | --- | --- | --- | --- | --- | --- | --- | --- | --- | --- | --- | --- | --- | --- | --- | --- | --- | --- | --- | --- | --- | --- | --- | --- | --- | --- | --- | --- | --- | --- | --- | --- | --- | --- | --- | --- | --- | --- | --- | --- | --- | --- | --- | --- | --- | --- | --- | --- | --- | --- | --- | --- | --- | --- | --- | --- | --- | --- | --- | --- | --- | --- | --- | --- | --- | --- | --- | --- | --- | --- | --- | --- | --- | --- | --- | --- | --- | --- | --- | --- | --- | --- | --- | --- | --- | --- | --- | --- | --- | --- | --- | --- | --- | --- | --- | --- | --- | --- | --- | --- | --- | --- | --- | --- | --- | --- | --- | --- | --- | --- | --- | --- | --- | --- | --- | --- | --- | --- | --- | --- | --- | --- | --- | --- | --- | --- | --- | --- | --- | --- | --- | --- | --- | --- | --- | --- | --- | --- | --- | --- | --- | --- | --- | --- | --- | --- | --- | --- | --- | --- | --- | --- | --- | --- | --- | --- | --- | --- | --- | --- | --- | --- | --- | --- | --- | --- | --- | --- | --- | --- | --- | --- | --- | --- | --- | --- | --- | --- | --- | --- | --- | --- | --- | --- | --- | --- | --- | --- | --- | --- | --- | --- | --- | --- | --- | --- | --- | --- | --- | --- | --- | --- | --- | --- | --- | --- | --- | --- | --- | --- | --- | --- | --- | --- | --- | --- | --- | --- | --- | --- | --- | --- | --- | --- | --- | --- | --- | --- | --- | --- | --- | --- | --- | --- | --- | --- | --- | --- | --- | --- | --- | --- | --- | --- | --- | --- | --- | --- | --- | --- | --- | --- | --- | --- | --- | --- | --- | --- | --- | --- | --- | --- | --- | --- | --- | --- | --- | --- | --- | --- | --- | --- | --- | --- | --- | --- | --- | --- | --- | --- | --- | --- | --- | --- | --- | --- | --- | --- | --- | --- | --- | --- | --- | --- | --- | --- | --- | --- | --- | --- | --- | --- | --- | --- | --- | --- | --- | --- | --- | --- | --- | --- | --- | --- | --- | --- | --- | --- | --- | --- | --- | --- | --- | --- | --- | --- | --- | --- | --- | --- | --- | --- | --- | --- | --- | --- | --- | --- | --- | --- | --- | --- | --- | --- | --- | --- | --- | --- | --- | --- | --- | --- | --- | --- | --- | --- | --- | --- | --- | --- | --- | --- | --- | --- | --- | --- | --- | --- | --- | --- | --- | --- | --- | --- | --- | --- | --- | --- | --- | --- | --- | --- | --- | --- | --- | --- | --- | --- | --- | --- | --- | --- | --- | --- | --- | --- | --- | --- | --- | --- | --- | --- | --- | --- | --- | --- | --- | --- | --- | --- | --- | --- | --- | --- | --- | --- | --- | --- | --- | --- | --- | --- | --- | --- | --- | --- | --- | --- | --- | --- | --- | --- | --- | --- | --- | --- | --- | --- | --- | --- | --- | --- | --- | --- | --- | --- | --- | --- | --- | --- | --- | --- | --- | --- | --- | --- | --- | --- | --- | --- | --- | --- | --- | --- | --- | --- | --- | --- | --- | --- | --- | --- | --- | --- | --- | --- | --- | --- | --- | --- | --- | --- | --- | --- | --- | --- | --- | --- | --- | --- | --- | --- | --- | --- | --- | --- | --- | --- | --- | --- | --- | --- | --- | --- | --- | --- | --- | --- | --- | --- | --- | --- | --- | --- | --- | --- | --- | --- | --- | --- | --- | --- | --- | --- | --- | --- | --- | --- | --- | --- | --- | --- | --- | --- | --- | --- | --- | --- | --- | --- | --- | --- | --- | --- | --- | --- | --- | --- | --- | --- | --- | --- | --- | --- | --- | --- | --- | --- | --- | --- | --- | --- | --- | --- | --- | --- | --- | --- | --- | --- | --- | --- | --- | --- | --- | --- | --- | --- | --- | --- | --- | --- | --- | --- | --- | --- | --- | --- | --- | --- | --- | --- | --- | --- | --- | --- | --- | --- | --- | --- | --- | --- | --- | --- | --- | --- | --- | --- | --- | --- | --- | --- | --- | --- | --- | --- | --- | --- | --- | --- | --- | --- | --- | --- | --- | --- | --- | --- | --- | --- | --- | --- | --- | --- | --- | --- | --- | --- | --- | --- | --- | --- | --- | --- | --- | --- | --- | --- | --- | --- | --- | --- | --- | --- | --- | --- | --- | --- | --- | --- | --- | --- | --- | --- | --- | --- | --- | --- | --- | --- | --- | --- | --- | --- | --- | --- | --- | --- | --- | --- | --- | --- | --- | --- | --- | --- | --- | --- | --- | --- | --- | --- | --- | --- | --- | --- | --- | --- | --- | --- | --- | --- | --- | --- | --- | --- | --- | --- | --- | --- | --- | --- | --- | --- | --- | --- | --- | --- | --- | --- | --- |

*Appendix S1: Search Keywords and Search Strings*

Embase 1974 to present:

1. Postnatal depression/
2. ((postpartum or postnatal or antepartum or antenatal or post-partum or post-natal) and depression).ti,ab.
3. 1 or 2
4. “systematic review”.ti,ab
5. 3 and 4

MEDLINE 1946 to present:

1. Depression, Postpartum/
2. ((postpartum or postnatal or antepartum or antenatal or post-partum or post-natal) and depression).ti,ab.
3. 1 or 2
4. “systematic review”.pt.
5. “systematic review”.ti,ab.
6. 8 or 9
7. 3 and 6
